# Dramatic response of CTNNB1 and VEGFR-2 mutant temporal bone squamous cell carcinoma to bevacizumab in combination with pemetrexed

**DOI:** 10.18632/oncotarget.19649

**Published:** 2017-07-28

**Authors:** Lai Wei, Lizhi Wang, Ziye Liu, Meiyi Wang, Weili Lu, Dewei Zhao, Bin Yang, Xuejun Kong, Yan Ding, Zhiqiang Wang

**Affiliations:** ^1^ Department of Otolaryngology, Affiliated Zhongshan Hospital of Dalian University, Dalian, China; ^2^ The Institute for Translational Medicine, Affiliated Zhongshan Hospital of Dalian University, Dalian, China; ^3^ Department of Pediatrics, Children’s Hospital of Boston, Harvard Medical School, Boston, MA, USA; ^4^ Program for Autism Research, Massachusetts General Hospital, Harvard Medical School, Boston, MA, USA

**Keywords:** temporal bone squamous cell carcinoma, recurrent tumor, targeted therapy

## Abstract

High recurrence rates and poor survival rates for late stage/advanced temporal bone squamous cell carcinoma with the standard treatments continues to be a significant challenge to otolaryngologists. Targeted therapy for temporal bone squamous cell carcinoma after relapse has not been reported. Here we present a 58-year-old man who was diagnosed with recurrent temporal bone squamous cell carcinoma and treated with a regimen developed using whole exome sequencing. Somatic mutations in genes encoding catenin beta 1 and vascular endothelial growth factor receptor 2 were identified in the patient’s tumor sample compared to the normal tissue. The patient was then treated with Bevacizumab in combination with pemetrexed. After two weeks of treatment, tumor volume was reduced by 95% measured by MRI, and the Visual Analogue Scale headache scores went down from 10/10 to 2/10. Our results reveal novel gene mutations of temporal bone squamous cell carcinoma and demonstrate, for the first time, an effective targeted therapy for temporal bone squamous cell carcinoma. The successful treatment regimen of bevacizumab and pemetrexed may provide a new treatment option for treating recurrent temporal bone squamous cell carcinoma that fails to respond to conventional tumor resection, radiotherapy, and/or chemotherapy.

## INTRODUCTION

Temporal bone squamous cell carcinoma (TBSCC) is uncommon, accounting for fewer than 0.2% of all tumors of the head and neck with an incidence of 1 to 6 per one million [1]. Only 200 new cases of temporal bone cancer may be diagnosed each year across the United States. This number includes cancers arising from skin of the pinna that spread to the temporal bone; primary tumors of the external auditory canal (EAC), middle ear, mastoid, or petrous apex; and metastatic lesions to the temporal bone. TBSCC accounts for 80% of all temporal bone tumors [2]. Formulating an optimal evaluation and treatment protocol for TBSCC continues to be a significant challenge to otolaryngologists due to its rare incidence and the complexity of the anatomy in the region [3]. Surgical resection combined with postoperative radiation therapy has been described as the standard of care for primary site TBSCC [4]. However, recurrence rate of a late stage aggressive subtype TBSCC is significantly high [5]. Recently, targeted therapy has been successfully used in a number of different cancers such as cervical cancer, non-small cell lung cancer, olfactory neuroblastoma, and head & neck cancers [6–11]. However, to the best of our knowledge, targeted therapy for TBSCC based on individual genomic profile has never been reported.

In the present case, a patient diagnosed with TBSCC had received resection of left temporal bone followed by radiotherapy and chemotherapy. He presented with a recurrence of TBSCC three months after surgical treatment. Identification of genomic variations in the tumor tissue made via whole exome sequencing (WES) led to the development of a treatment regimen of bevacizumab combining with pemetrexed. We report herein the first exceptional therapeutic response to bevacizumab targeted therapy in combination with pemetrexed chemotherapy in a multiply recurrent TBSCC with genetically confirmed vascular endothelial growth factor receptor 2 (VEGFR-2) and catenin beta 1 (CTNNB1) mutation.

## CASE REPORT

A 58-year-old male was diagnosed with left maxillary sinus squamous cell carcinoma and underwent a surgical operation in September 2013. He complained of nasal obstruction, facial swelling and intermittent epistaxis three months later. Dark red neoplasm located in the patient left nasal cavity was observed. CT scan showed the invasion of multiple structures including anterior left frontal sinus, ethmoid sinus, maxillary sinus, nasal septum, pterygopalatine fossa and hard palate. The patient received tumor radical excision and confirmed to be squamous cell carcinoma by pathological examination.

After the surgery, the patient received radiotherapy of 70 Gy in fractions of 2 Gy. However, one year and five months after post-operative treatment, the patient presented with complaints of intermittent epistaxis and decreased vision of the left eye for two months. Examination showed that he had loss of vision and a tumor was located on the top wall of the left residual hard palate. The patient agreed to receive extended resection of the left maxilla. After that, he received radiotherapy of 50 Gy in fractions of 1.3Gy and 5 courses of chemotherapy, including paclitaxel (IV, once every three weeks, 240mg at a time) (135 mg/m2) and nedaplatin (IV, once every three weeks, 140mg at a time) (80 mg/m2). The complication of leukopenia was observed and it was recovered after using recombinant human granulocyte colony-stimulating factor. Oral ulcer healed, but hair losing because of early chemotherapy. Nausea and vomiting were disappeared after injection of antipathectic.

**Figure 1:**
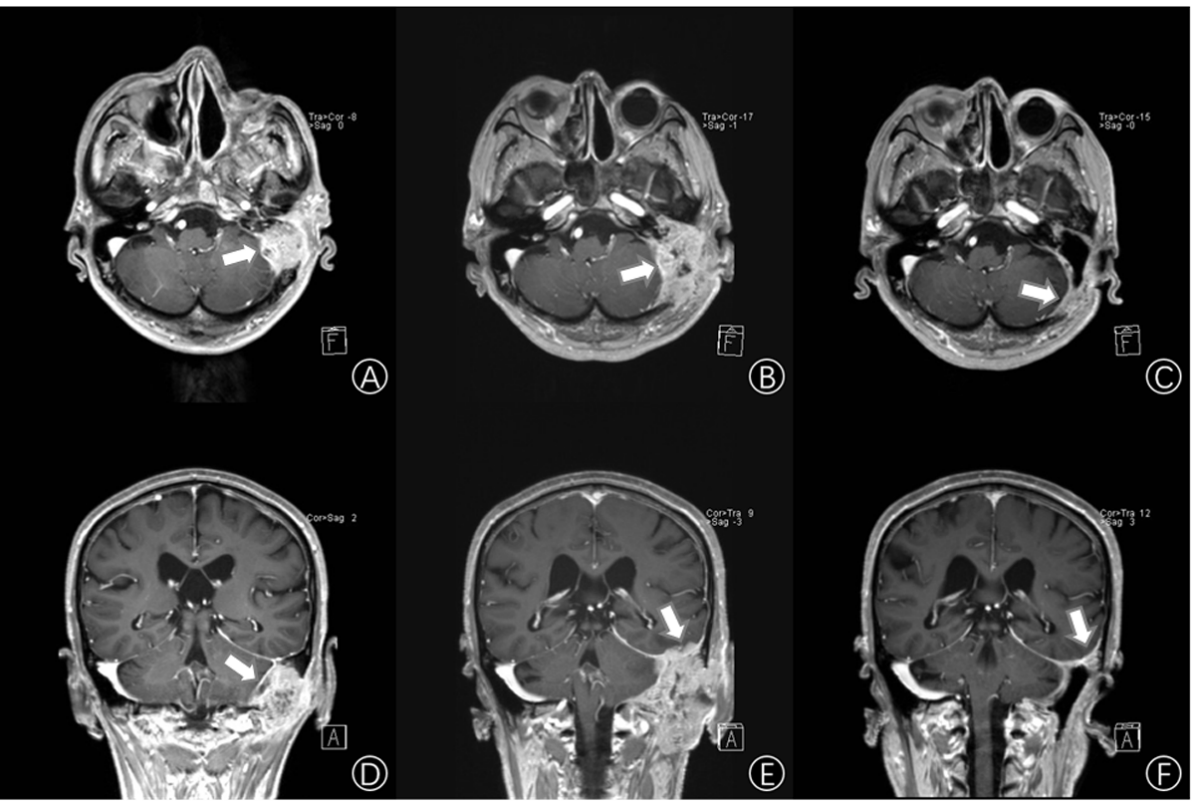
Imaging examination **A.** and **D.** Enhancement cerebral MRI showing the left temporal lobe space-occupying lesion (white arrows) (size:27.5mm x 38.0mm x 40.5mm). **B.** and **E.** Three months after the first middle ear surgery, enhancement cerebral MRI shows the tumor (white arrows) is recurrent (size:40.5mm x 52.3mm x 63.6mm). **C.** and **F.** Two weeks after bevacizumab and pemetrexed treatment, an enhancement cerebral MRI shows that the right temporal lobe space occupying lesion has reduced by 95% (size: 13.2mm x 20.5mm x 27.0mm). (A, B, C. Horizontal enhancement imaging. D, E, F. Coronal enhancement imaging).

**Figure 2:**
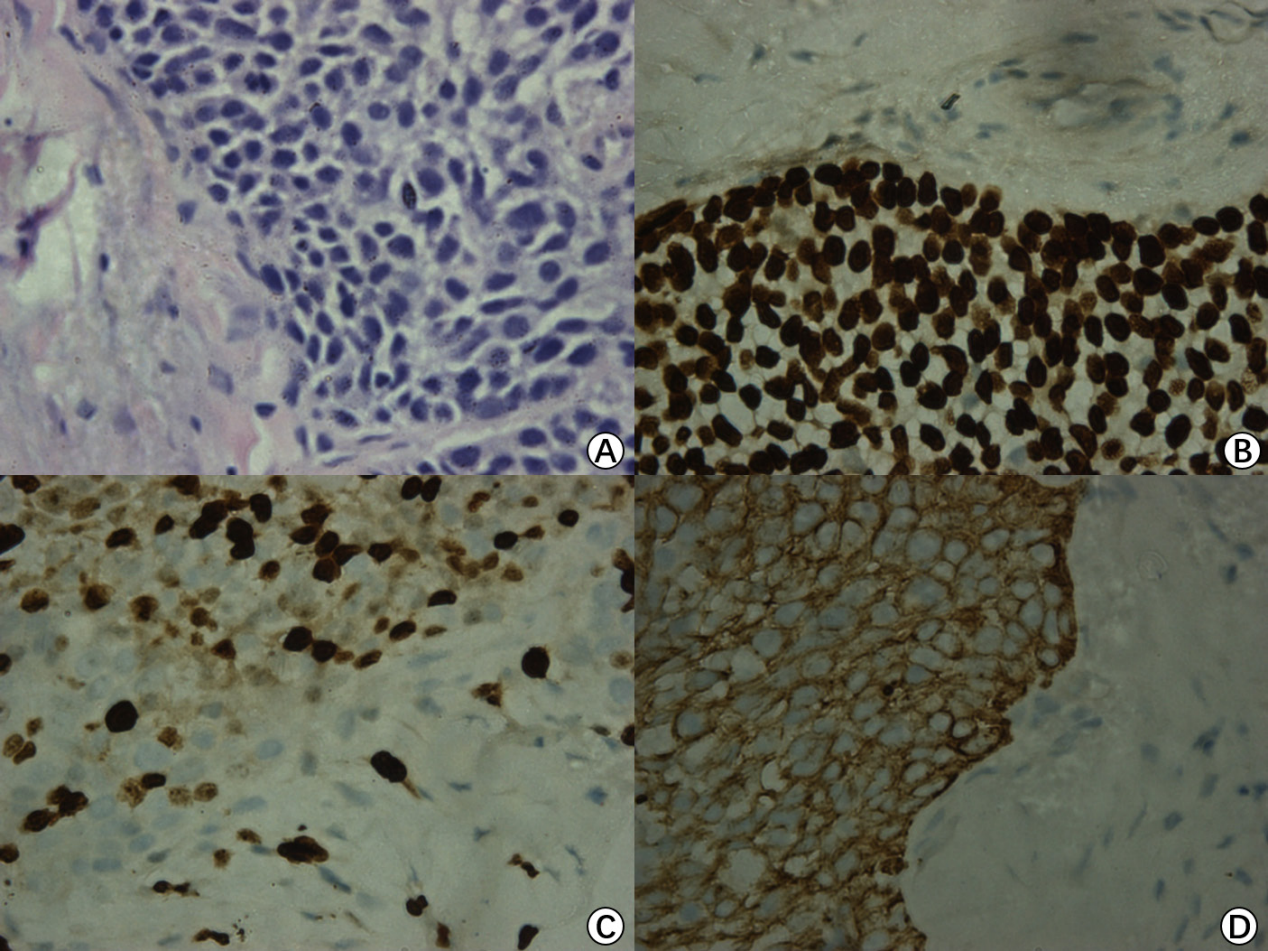
Pathological examination H&E staining (×400) showed some pink cytoplasm with distinct cell borders and intercellular bridges characteristic **A.** Immunohistochemical results show thatP63 **B.**, Ki67 **C.** and CK516 **D.** are positive (×400).

Three years after the first surgery, the patient came back and complained of hearing loss and otorrhea for 10 months. White neoplasm located at the patient left EAC was observed. CT scans showed the invasion of multiple structures including EAC, tympanic cavity, mastoid and occipital. MRI distinctly showed that there was a lesion occupying the left temporal bone nearby the left middle fossa of skull (size:27.5mm x 38.0mm x 40.5mm, T4) (Figure 1A, 1D). The tumor tissues were confirmed as TBSCC by using H&E staining and Immunohistochemistry (Figure 2).

Then, after radical resection of the tumor for 3 months, it recurred and invaded the left cerebella hemisphere and temporal lobe (size:40.5mm x 52.3mm x 63.6mm, T4) by MRI scan (Figure 1B, 1E). That made removing the recurrent tumor more difficult and risky.

Given the failure of tumor resection, we decided to explore the options for targeted therapy for this patient. WES was employed to detect the target gene mutations from patient tumor/normal tissue pairs on the Illumina NextSeq500 sequencing platform and using a TruSeq Rapid Capture Exome Kit for library construction. The WES data was then analyzed using OncoDecoderTM (Genomic Future, Inc.). The significantly mutated cancer-related genes that were identified in the tumor tissue compared to the adjacent normal control included CTNNB1 and VEGFR-2 (Table 1).

**Table 1:** The Significantly Mutated Genes from the Tumor Tissue of the Patient Detected by Whole Exome Sequencing

Gene name	Exon	Amino Acid Change	Nucleotide Change	Genotype	Mutation type
CTNNB1	3th	p.Met12Leu	c.34A>C	heterozygosis	Missense mutation
VEGFR-2	11th	p.Gln472His	c.1416A>T	homozygosis	Missense mutation

**Figure 3:**
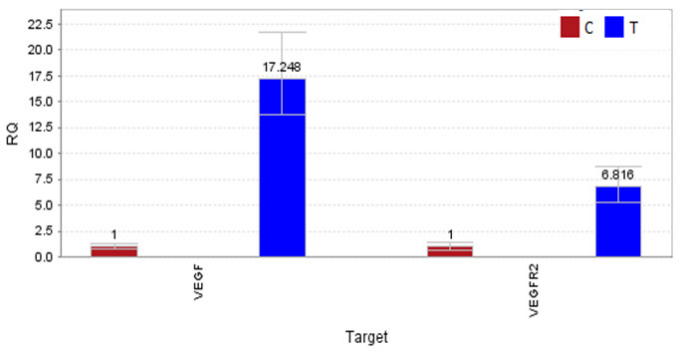
The result of quantitative polymerase chain reaction (qPCR) Overexpression of VEGF and VEGFR-2 in TBSCC tissue compared to normal tissue by qPCR. Expression of VEGF is about 17-fold and VEGFR-2 is about 7-fold higher than normal tissues.

Specifically, we found a missense mutation p.Met12Leu in exon3 of CTNNB1 and missense mutation p.Gln472His(exon11) in VEGFR-2. To detect the gene expression level of VEGFR-2 and its ligand vascular endothelial growth factor(VEGF), quantitative real time PCR on mRNA extracted from both tumor and normal tissues was carried out, and results showed significant overexpression of both VEGF and VEGFR-2 genes in the tumor tissue compared to the normal tissue (Figure 3).

Based on these findings, we carefully screened currently available targeted drugs that may potentially be used for treatment of head and neck cancers. We determined a treatment regimen of bevacizumab and pemetrexed. The ethics committee of the Affiliated Zhongshan Hospital of Dalian University approved the study (The Institutional Review Board approval number is 2016102). The patient received concurrent Bevacizumab (IV, one time per two weeks, 300mg each time for 2 weeks) treatment and pemetrexed (IV, one time a day, 890mg per 21 days for 4 weeks) treatment. The patient was found in slightly red erythra with itch, and these symptoms resolved spontaneously within 48 hours. Fifteen days later, MRI revealed that the lesion occupying the left temporal lobe space had significantly shrunk by 95% to a small hypointense area (size13.2mm x 20.5mm x 27.0mm, T4) (Figure 1C, 1F). Visual Analogue Scale scores of headaches were from 10/10 to 2/10. On targeted therapy Day 35, the patient received extended resection of the temporal bone and reconstruction. Two months later, the patient remains free of new symptoms. Long-term follow-up care has been established for the patient.

## DISCUSSION

A combination of open surgery, radiotherapy and/or chemotherapy has been considered as standard care for TBSCC. However, it was deemed to have a negative outcome and prognosis. Dean NR et al. reported that the rate of recurrence in TBSCC is close to 34% [12].

Takenaka, Y et al. reported that Paclitaxol, Cisplatin and 5-fluorouracil are some of the common chemotherapeutic medicine used in squamous cell carcinoma of the EAC [4]. Unfortunately, the traditional treatment regimens often fail for recurrent and metastatic TBSCC cases, especially with stage T3-4 [13]. Moreover, some patients may refuse to take the standard protocols due to their intolerance of radiation and/or chemotherapy. Most recently, genome-based precision medicine has drawn a great deal of attention from oncologists. Several studies have shown that the use of targeted medicines can result in either a complete response or a significant improvement in the life quality of patients with a variety of cancers [16]. It is widely accepted that one of the biggest advantages of targeted drugs is their high specificity and low toxicity. To the best of our knowledge, personalized treatment of TBSCC with targeted drugs based on the patient’s genomic variations has not been reported.

To explore the opportunity and potential benefit of targeted therapy for advanced and recurrent TBSCC, we sequenced the whole exomes of the tumor/normal tissues of the patient in our genetic test lab and identified several novel significantly mutated cancer genes that may be related to TBSCC including CTNNB1 and VEGFR-2 (Table 1). A few of gene mutation and/expression dysregulation including p16, TP53 mutation, epidermal growth factor receptor (EGFR), pSTAT3, and relaxin-2 have been reported that may be related to the development or progression of different categories of TBSCC [14]. For example, studies have shown overexpression of p53 and EGFR may be valuable biomarkers for identifying TBSCC with high risk of lymph node metastasis [15]. However, in our case, we found novel mutations in CTNNB1 and VEGFR-2 genes that may be related to TBSCC and overexpression of VEGF and VEGFR-2 genes were observed in the tumor tissue. VEGFR-2, also known as vascular endothelial growth factor receptor 2, functions as the main mediator of VEGF-induced angiogenesis in a variety of cancers. Bevacizumab, an anti-vascular endothelial growth factor monoclonal antibody, was the first anti-angiogenesis agent to receive US Food and Drug Administration approval in oncology [16]. Numerous studies of the bevacizumab showed promise in patients with squamous cell carcinoma of the head and neck (SCCHN) [17, 18]. Nylfot, MJ et al. reported that bevacizumab could reduce tumor proliferative capacity of SCCHN, and combined bevacizumab with cisplatin-based chemo-radiation therapy for SCCHN revealed safe and significant responses [19]. Pemetrexed disodium is a structurally modified folate analogue [20]. It has proven effective in non-small-cell lung cancer and malignant pleural mesothelioma that suppresses both DNA synthesis and folate metabolism [21–23]. In 2011, a result of 40 patients research showed that adding bevacizumab to pemetrexed resulted in promising efficacy outcomes in SCCHN [24]. In our case, genetic tests have shown mutation in VEGFR-2 (Table 1) as well as significant overexpression of VEGFR-2 and VEGF in the patient TBSCC tumor tissue (Figure 3). Since bevacizumab may indirectly blocks VEFGR-2 signaling through inhibiting VEGF, it is perhaps not surprising to see that the patient showed a striking response to a bevacizumab plus pemetrexed treatment regimen (Figure 1). The clinical outcomes from the TBSCC case indicate that VEGFR-2 might be potential target for TBSCC therapy.

TBSCC is an aggressive malignancy with a poor prognosis in advanced cases. The 5-year survival rate for patient with T3-4 was 14.0%-55.6% [13, 25]. T4 classification, extensive bone involvement and dura involvement was poor predictor of survival rate, separately [26]. Two months after receiving extended resection of the temporal bone and reconstruction, the patient remains free of new symptoms. Shrinking tumors before definitive treatment with surgery/radiation could improve the safety and efficacy of initial treatment, potentially reducing the disabling morbidities that often follow current treatment. As we prepared the final draft of out manuscript, the patient remains in stable state. We recognized the limitations of our study were based on single and short time follow-up case, therefore, it is not clear at this point of time that the targeted therapy would prolong the overall survival of the rare malignant tumor.

CT of the temporal bone is the standard for assessing bone erosion, while MRI, provides detailed soft tissue resolution, is superior to CT in diagnosis of temporal bone malignancy that invades the intracranial space and brain tissue and evaluation of the major blood vessels of the skull base. In this case, the patient only receives MRI scanning after radical resection of the malignancy because of the hazards of CT scanning and the advantages of MRI.

Follow-up care has been established for the patient and an update of clinical outcomes is warranted. As TBSCC is a rare disease, collaborative efforts for a cohort study is desirable to further prove the effectiveness of targeted therapy for TBSCC.

## CONCLUSIONS

Surgery followed by chemo-radiotherapy is the current standard of care for TBSCC and fails frequently with a high risk of recurrence and adverse effects. Genome-based targeted therapy for recurrent and late stage TBSCC is potentially an option in terms of its promising clinical response, and hence deserves further investigation in a prospective clinical trial.

## Abbreviations

TBSCC: Temporal bone squamous cell carcinoma
WES: whole exome sequencing
CTNNB1: catenin beta 1
EAC: external auditory canal
VEGFR-2: vascular endothelial growth factor receptor 2
VEGF: vascular endothelial growth factor
EGFR: epidermal growth factor receptor
SCCHN: squamous cell carcinoma of the head and neck
